# Right Prefrontal Activity Reflects the Ability to Overcome Sleepiness during Working Memory Tasks: A Functional Near-Infrared Spectroscopy Study

**DOI:** 10.1371/journal.pone.0012923

**Published:** 2010-09-23

**Authors:** Motoyasu Honma, Takahiro Soshi, Yoshiharu Kim, Kenichi Kuriyama

**Affiliations:** Department of Adult Mental Health, National Institute of Mental Health, National Center of Neurology and Psychiatry, Tokyo, Japan; Cuban Neuroscience Center, Cuba

## Abstract

It has been speculated that humans have an inherent ability to overcome sleepiness that counteracts homeostatic sleep pressure. However, it remains unclear which cortical substrate activities are involved in the ability to overcome sleepiness during the execution of cognitive tasks. Here we sought to confirm that this ability to overcome sleepiness in task execution improves performance on cognitive tasks, showing activation of neural substrates in the frontal cortex, by using a modified *n*-back (2- and 0-back) working memory task and functional near-infrared spectroscopy. The change in alertness was just correlated with performances on the 2-back task. Activity in the right prefrontal cortex changed depending on alertness changes on the 2- and 0-back tasks independently, which indicates that activity in this region clearly reflects the ability to overcome sleepiness; it may contribute to the function of providing sufficient activity to meet the task load demands. This study reveals characteristics of the ability to overcome sleepiness during the n-back working memory task which goes beyond the attention-control function traditionally proposed.

## Introduction

Humans may inherently possess the ability to overcome sleepiness when faced with completing assignments accurately or within a given time frame [1], [2]. Heightened ability to overcome sleepiness, which may be driven by heightening cortical availability for accelerating cognitive performance [3], [4], enables them to do such assignments more accurately or quickly [5]. When the same cognitive task was loaded under similar sleep pressure, some individuals could carry out the task adequately by overcoming the sleep pressure, while others could not. A large body of research describes the effects of sleep loss on human cognitive performance [6], [7]. Sleep loss not only through the entire night but also for a few hours in a night can deteriorate certain aspects of cognitive performance [8], [9]. Total sleep deprivation (TSD) partially extinguishes physiological recovery normally gained through sleep, in respect to both homeostatic property and circadian regulation, and the impairments to cognition seen after TSD appear to be a consequence of the overall effect of such physiological dysfunction [10]. On the other hand, sleep loss of several hours, which can occur on a daily basis, does not cause serious physiological dysfunction, although some cognitive impairments often result [11]. Sadeh et al. [12] elucidated that such impairments might be caused by accumulated sleepiness due to sleep restriction, with some cognitive functions being vulnerable to accumulated sleepiness. Although cognitive impairments caused by TSD are a topic of major importance among shift workers, cognitive impairments caused by slight sleep loss often occur and constitute a universal phenomenon in daily life.

The effects of sleep loss on various cognitive functions have been elucidated by using a simple reaction task [13], [14] and a working memory (WM) task [15] in both healthy and sleep disordered subjects [16]. WM is a cognitive process dedicated to the transitory maintenance and online manipulation of information [17] applied in a broad range of higher cognitive functions including comprehension, reasoning, planning and learning [18], and is an outcome of the ability to control and sustain attention and to focus it strategically on a particular mental representation in the face of distracting influences [19]. Deterioration in alertness caused by sleep loss induced by not only TSD [10] but also partial sleep deprivation [20] generally leads to a decline in WM performance [15], which occurs as a consequence of decrements in prefrontal cortex (PFC) activity [21], [22].

Some behaviors for conquering sleepiness have been reported to be able to improve WM performance and the responsible neural activities. Chewing, which has potential alerting effects [23], [24], enhanced activities in the bilateral dorsolateral prefrontal cortex (DLPFC) during a WM task [25]. Smoking a cigarette also enhanced left DLPFC activity during such a task [26]. Given the psychopharmacological effects of nicotine, which stimulates dopaminergic neural activity in the midbrain [27], the rapid enhancement effect for DLPFC activity suggests that smoking itself could directly accelerate WM task performance, even if alerting effect also collaterally works. One of the important roles of the DLPFC in WM is reported to be attention control [28], which is the ability to focus on task-relevant goals to the exclusion of salient distracters. The alerting effect appears to heighten this ability for improved WM performance.

We hypothesized the following: the ability to overcome sleepiness might enhance WM task performance, helping to resist sleepiness in efforts to carry out a task; the ability to overcome sleepiness might depend on DLPFC activity, which may be shared with attention-control ability; and the ability to overcome sleepiness might show inter-individual differences.

To verify these hypotheses, we used a modified *n*-back WM task with two separate load levels [29], [30] to confirm whether inter-individual differences in the ability to overcome sleepiness exist in task execution. In order to distinguish individuals by their ability to overcome sleepiness, subjective alertness in the pre- and post-WM task periods was compared. Further, we focused on changes in oxygenated hemoglobin (oxy-Hb) in the prefrontal region in relation to WM performance by using functional near-infrared spectroscopy (fNIRS) [31]. fNIRS has been commonly applied to the study of prefrontal activity [32], [33] and is suitable for characterizing oxygenation changes in higher cortical regions during cognitive tasks. It is also well suited to sleep restriction studies because it does not produce high noise levels on scanning or require that subjects severely restrict their body movement, and thus is unlikely to seriously influence the sleep-restricted condition [34].

## Methods

### Subjects

**Figure 1:**
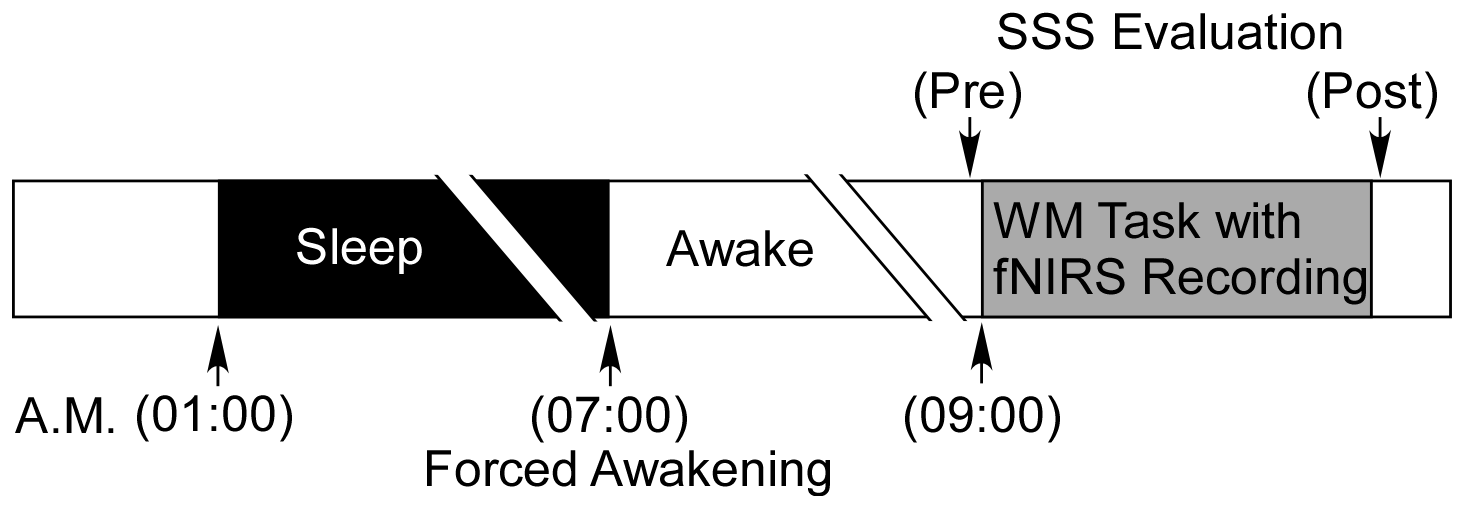
Experimental protocol. Subjects retired to bed at 01:00 A.M. in the laboratory and were forcibly awakened at 07:00 A.M. by an experimenter. The average duration of actual sleep period was 5.53 h (SEM: 0.05 h). Subjects started working memory (WM) tasks with f-NIRS acquisition 2 h after forced awakening at 09:00 A.M. and were kept awake before the WM tasks commenced. Subjective alertness level was evaluated immediately before and after the experiment using the Stanford Sleepiness Scale (SSS). WM tasks took between 15 to 30 minutes to complete.

Fifty-five right-handed healthy volunteers participated in this study (29 female; mean age, 21.2 years; range, 19–26 years). Subjects completed a self-assessment questionnaire about their sleep habits in daily life. From the data obtained, habitual awakening time was 07:00–09:00 (average: 08:37, SEM: 0.12 h), habitual sleep onset was 23:00–01:00 (average: 00:51, SEM: 0.11 h) and the average habitual sleep duration was 7.86 h (SEM: 0.11 h), as confirmed by a sleep log over a 3-day period prior to the experiment. Subjects participated in an overnight experiment in a laboratory setting, starting at 22:00 on day 1 and finishing at 10:00 on day 2 (see Fig. 1). The experimental procedure was in accordance with the Declaration of Helsinki and was reviewed and approved by the Ethics Committee of the Intramural Research Board of the National Center of Neurology and Psychiatry. All volunteers gave their written informed consent after receiving a full explanation of the procedures involved.

### Sleep restriction

All subjects retired to bed at 01:00 A.M. in the laboratory and were awakened immediately at 07:00 A.M. by an experimenter. The average sleep duration was 5.53 h (SEM: 0.05 h), as confirmed by using an ambulatory wrist activity recorder (Actiwatch-L, Mini-Mitter co., Inc. Bend, OR). Subjects had a restricted sleep duration of 2.32 h (SEM: 0.07 h) on average (29.5%). To avoid the effect of sleep inertia [35], [36], the experiment started 2 h after rising and the subjects were kept awake during this interval.

### Alertness level

Subjective alertness level was evaluated immediately before and after the experiment using the Stanford Sleepiness Scale (SSS) [37]. The SSS is one of the most frequently employed scales to assess subjective alertness level and consists of a 7-point scale ranging from level 1 (feeling active, vital, alert or wide awake) to level 7 (no longer fighting sleep). Subjects selected the most appropriate level to reflect their present state of alertness. To assess individual ability to overcome sleepiness during WM tasks, change in SSS level was individually calculated by subtracting the post-experiment SSS score from the pre-experiment SSS score.

### Working memory task

The cognitive paradigm employed in the present study consisted of a visual *n*-back WM task with two separate load levels. For the 0-back task (low-load WM task), subjects were instructed to press a response button using their dominant hand whenever a single-digit number appeared on a 20-inch LCD computer screen. For the 2-back task (high-load WM task), they had to press a button on the right with their middle finger when the single-digit number on the screen was identical to that which had appeared last but one, otherwise to press the button on the left with their index finger. A 1500-ms baseline period preceded all the tasks. Numbers were presented in randomized order for 300 ms with a 1700-ms inter-stimulus interval. Each level of task was run in blocks of 12+n stimuli and was conducted two times; thus, 24 responses were obtained at each load level. The inter-trial interval was set at 2500 ms. From the performance data recorded, average response times (RTs) and correct response rates (%CRs) were determined.

### fNIRS data acquisition

**Figure 2:**
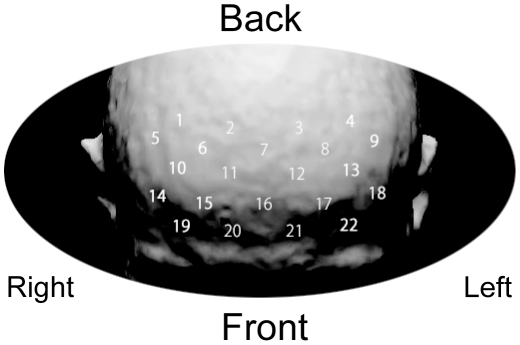
Approximate position of the probe holder. The distance between the pair of emission and detector probes was 3.0 cm, and it was considered that the machine could measure points at a depth of 2–3 cm from the scalp. A 3×5 (6×12 cm) probe holder was placed on the scalp surface covering the bilateral prefrontal cortex. The measurement points were labeled channels 1 to 22 from right-side top to left-side bottom.

Changes in [oxy-Hb] and [deoxy-Hb] concentration were continuously measured with a time resolution of 0.1 s using a 22-channel fNIRS machine (ETG-100, Hitachi Medical Corp., Tokyo) at two wavelengths of near-infrared light (i.e., 780 and 830 nm), and [oxy-Hb] and [deoxy-Hb] levels were calculated based on the modified Beer-Lambert equation as a function of light absorbance of Hb and path length. The distance between the pair of emission and detector probes was 3.0 cm, and it was considered that the machine could measure points at a depth of 2–3 cm from the scalp (Fig. 2), that is, at the surface of the cerebral cortex [38]–[40]. A 3×5 (6×12 cm) probe holder was placed on the scalp surface covering the bilateral PFC with the lowest central edge of the holder positioned along the Fp1–Fp2 line according to the international 10/20 system used in electroencephalography. The measurement points were labeled channels 1 to 22 from right-side top to left-side bottom. The correspondence between the recording sites and the bilateral middle and superior PFC was previously confirmed by a multisubject study of anatomical craniocerebral correlation [41].

### fNIRS data analysis

We analyzed oxy-Hb data as a reflection of event-related responses in the PFC because change in oxy-Hb concentration is thought to be the most reliable index of regional cerebral blood flow. The continuous oxy-Hb data were filtered with band-pass frequencies in the range of 0.01–0.2 Hz and were standardized (z-score) in order to avoid the methodological ambiguity that changes in absolute values of Hb concentration for each recording channel were not determined, because the absolute path lengths of light through the cerebral cortex were not detectable. Changes in oxy-Hb concentration time-locked to the experimental blocks consisting of 12 trials were extracted for each experimental condition 1000 ms before the onset of the trial block (baseline) to 4400 ms after the onset (2400 ms for the 12-trial duration and 2000 ms for the post-trial interval), which amounted to a total duration of 5400 ms. Baseline correction of the changes in oxy-Hb concentration was performed utilizing the mean z-scores of 1000 ms prior to the onset of the experimental blocks before individual averaging. Mean z-scores of the changes in oxy-Hb concentration during the 4400 ms period after the start of the trial block were utilized for subsequent statistical analyses.

### Statistics

To examine the difference in alertness between the pre- and post-experiment, paired-t tests were performed for SSS scores. To examine the difference in WM performance between task levels, paired-t tests were performed for RTs and %CR. To examine the relation between effects of overcoming sleepiness and WM performance, we analyzed Pearson's correlation coefficients of change in SSS level and %CR or RTs for each WM task level separately. To estimate the effects of overcoming sleepiness on the prefrontal activities corresponding to WM characteristics, we analyzed Pearson's coefficients between change in SSS level and the difference of change in oxy-Hb concentration for each channel on the 0- and 2-back tasks. To estimate the effects of overcoming sleepiness on the prefrontal activities corresponding to each WM task level separately, we analyzed Pearson's coefficients between change in SSS level and change in oxy-Hb concentration for each channel on the 0- and 2-back tasks separately.

## Results

### SSS score and *n*-back performance

A paired-t test revealed that the mean pre-experiment SSS score (average: 2.60, SD: 0.81) did not differ significantly from that of the mean post-experiment SSS score (average: 2.69, SD: 1.23) [t(54) = −0.647, p = 0.527]. Twelve of 55 subjects showed an increased SSS level (average: 1.41, SD: 0.41), 17 showed a decreased SSS level (average: −1.29, SD: 0.49), and 26 showed no change in SSS level post-experiment compared to pre-experiment.

**Figure 3:**
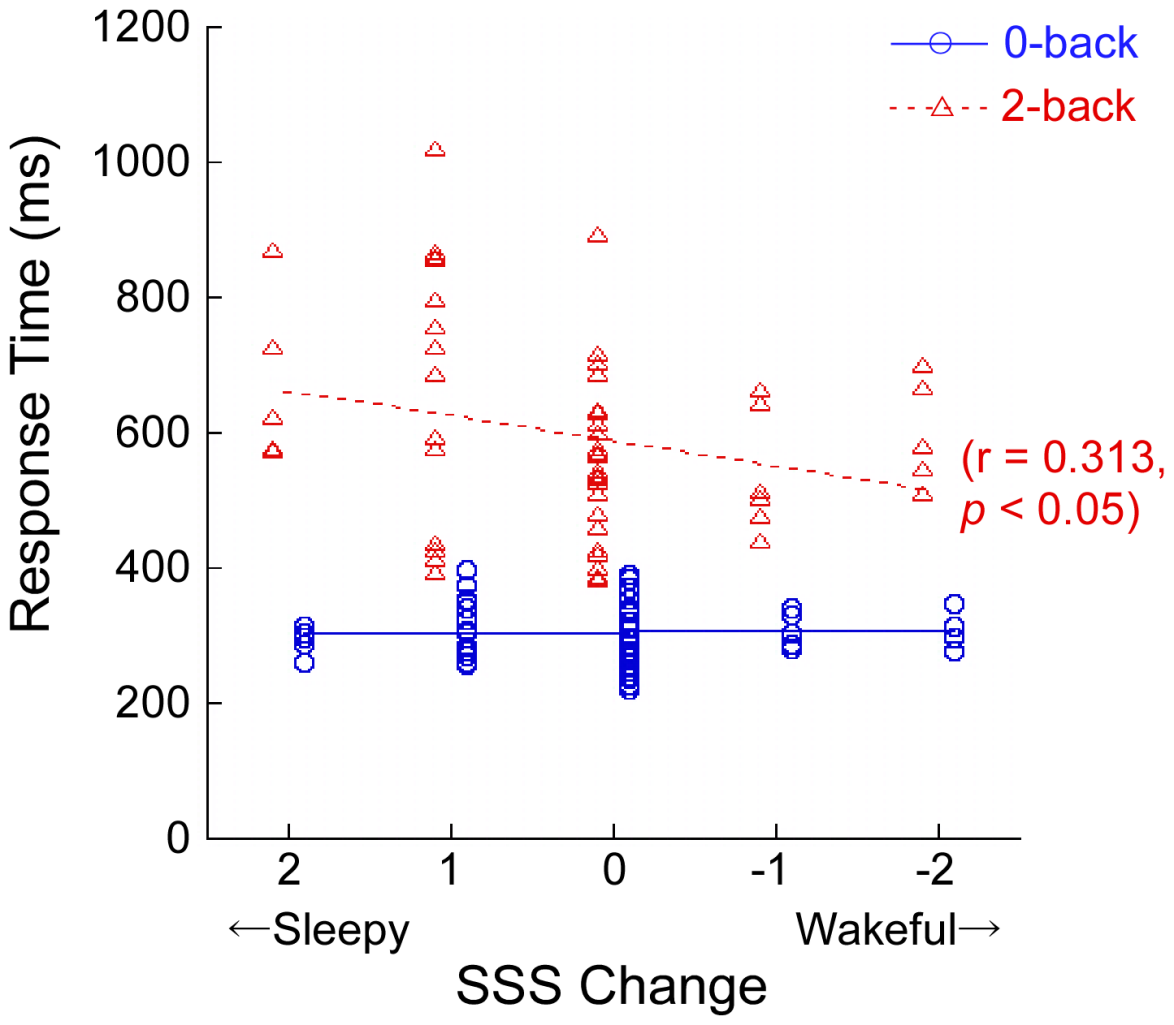
Correlation of change in Stanford Sleepiness Scale (SSS) level with reaction time. The horizontal line shows the difference in SSS level between the pre- and post-tasks. Positive values reflect the subject was awakened while performing the task. Blue circles denote 0-back task and red triangles denote 2-back task.

A paired-t test revealed that RT was significantly shorter on the 0-back task (average: 306 ms, SD: 53 ms) than on the 2-back task (average: 477 ms, SD: 72 ms) [t(54) = 115.941, p<0.0001], and %CR was higher on the 0-back task (average: 98.33%, SD: 1.68%) than on the 2-back task (average: 92.73%, SD: 5.21%) [t(54) = 5.816, p = 0.046]. Pearson's correlation coefficient revealed that the change in SSS level was negatively correlated with RT on the 2-back task (r = −0.313, p = 0.020), but not on the 0-back task (r = −0.089, p = 0.520) (Fig. 3), and the change in SSS was not correlated with %CR on either the 2-back (r = 0.223, p = 0.102) or 0-back (r = 0.100, p = 0.468) task.

### fNIRS data

**Figure 4:**
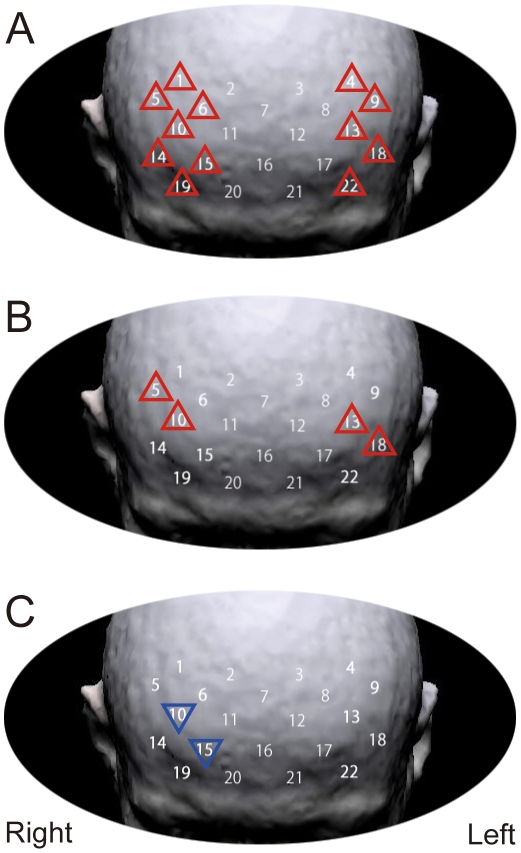
The results of fNIRS data. Numbers indicate the probes. Red upright triangles denote positive correlations and blue inverted triangles denote negative correlations. (A) Correlation of z-score and change in SSS level for the difference between 2- and 0-back tasks for each channel. (B) Correlation of z-score and change in SSS level for 22 channels on the 0-back task. (C) Correlation of z-score and change in SSS for 22 channels on the 2-back task.

**Figure 5:**
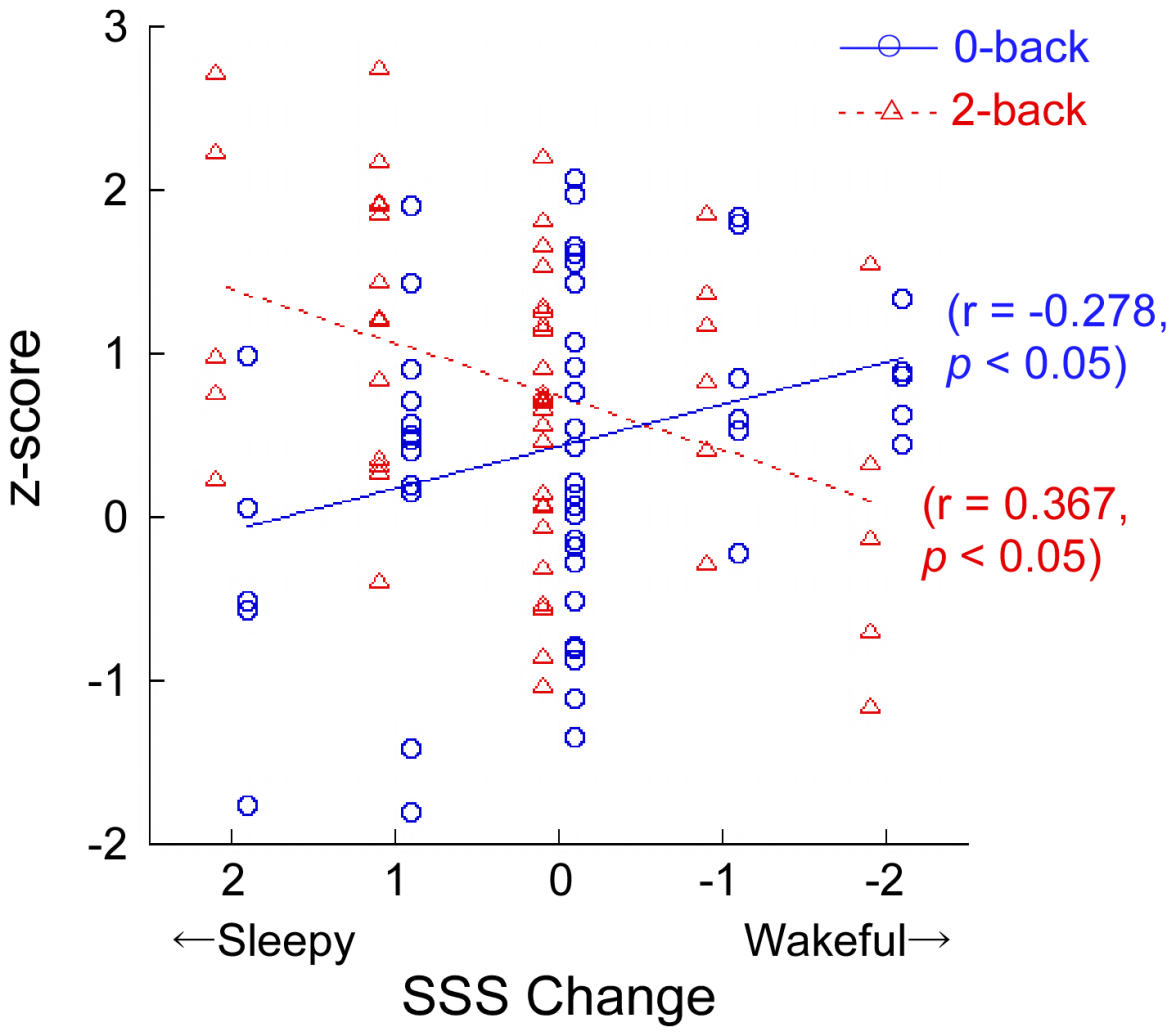
Correlation of z-score and change in SSS level on both the 2- and 0-back tasks for channel 10. Horizontal line shows the difference in SSS level pre- and post-experiment. Positive values reflect the subject was awakened while performing the task. Blue circles denote 0-back task and red triangles denote 2-back task.

Pearson's correlation coefficient revealed significant positive correlations between the difference of changes in oxy-Hb concentration on the 2- and 0-back tasks, and changes in SSS level were found for the left hemispheric channels of 4 (r = 0.301, p = 0.025), 9 (r = 0.271, p = 0.046), 13 (r = 0.279, p = 0.039), 18 (r = 0.381, p = 0.004) and 22 (r = 0.328, p = 0.014) and the right hemispheric channels of 1 (r = 0.289, p = 0.032), 5 (r = 0.367, p = 0.006), 6 (r = 0.295, p = 0.029), 10 (r = 0.489, p = 0.0001), 14 (r = 0.348, p = 0.009), 15 (r = 0.383, p = 0.004) and 19 (r = 0.324, p = 0.016) (Fig. 4A). Pearson's correlation coefficient between change in SSS level and change in oxy-Hb concentration for each channel for individual task levels showed positive correlations for channels 5 (r = 0.367, p = 0.006), 10 (r = 0.278, p = 0.040), 13 (r = 0.341, p = 0.011) and 18 (r = 0.402, p = 0.002) on the 0-back task (Fig. 4B), and negative correlations for channels 10 (r = −0.367, p = 0.006) and 15 (r = −0.313, p = 0.020) on the 2-back task (Fig. 4C). Only the change in oxy-Hb concentration for channel 10 was correlated to both the 2- and 0-back tasks (Fig. 5). Other channels showed no significant correlation (p≥0.05).

## Discussion

The subjects who participated in the experiment were confirmed to have similar sleep-wake habits in daily life and they experienced the same restricted sleep duration on the experimental night. However, in the post-task period, alertness was increased in some subjects but was decreased in others despite the subjects having performed the same tasks. Better RTs on the 2-back WM task were associated with the increase in alertness through performing the tasks. In contrast, however, the changes of alertness were not correlated with RTs on the 0-back task. Taken together, these findings indicate that those subjects who improved their alertness, who may have better abilities to overcome sleepiness, showed better performance on the high-load WM task, but this improved alertness did not contribute to better performance on the low-load WM task. However, as there was no significant correlation between %CRs and alertness changes on either the 2- or 0-back task, the RTs may better reflect the influence of the ability to overcome sleepiness upon performance on the current WM tasks. These results may contribute to our understanding of the individual differences in tolerance for shift work or sleep disturbance disorders [1], [2].

Changes in oxy-Hb concentration with WM load that correlated positively to changes in alertness were observed in the bilateral DLPFC, as suggested in previous fMRI studies [16], [25]. This finding clearly suggests that the correlation between alertness change and bilateral DLPFC activities encompasses the contrasting patterns that emerged in the correlation between changes in alertness and changes in oxy-Hb concentration on the 2-back and 0-back tasks. On the 2-back high-load task, neural substrates corresponding to channels 10 and 15 were deactivated in accordance with improved alertness, whereas on the 0-back low-load task, neural substrates corresponding to channels 5, 10, 13 and 18 were activated in accordance with improved alertness. Especially, the activity in channel 10 only showed opposite behavior corresponding with the 2-back and 0-back tasks, indicating that this region clearly reflects the ability to overcome sleepiness; it might contribute to the function of providing sufficient activity to meet the task load demands. In other words, the cortical activity corresponding to channel 10 susceptibly escalates with changes in both alertness and cognitive load and, as a consequence, better task performances are achieved. Previous research has reported that the region corresponding to around channel 10 covers the middle frontal gyrus in the right DLPFC [42], and this region might therefore be related to the ability to overcome sleepiness with cognitive performance.

Recent studies have reported that maintaining attention in the evening was associated with higher activity in the evening chronotype than in the morning chronotype [43], [44] in a region of the locus coeruleus and in the suprachiasmatic area [45]. A confounding factor has been suggested: that the effect of time-of-day which depends on individual traits of chronotype (i.e., morningness/eveningness tendencies) intervenes in the relation between sleep loss and cognitive performance. The sleep-wake habits of the current subjects were almost unified and did not meet the criteria of either morning or evening type on the Morningness-Eveningness questionnaire [46], and therefore we believe the current results represent the characteristics of the ability to overcome sleepiness that go beyond the chronotype. These results also suggest an individualized trait of right-DLPFC vulnerability to sleep loss while executing high-load WM tasks. The findings of Hamilton et al. [47] that subjects with bipolar disorder and schizophrenia show hyperactivity during a low-load WM task and subsequent deterioration of performance support our notion.

Finally, we recognize a limitation regarding the measurement of subjective sleepiness. Although we evaluated the relationship between task performance and subjective sleepiness, a more suitable measure for evaluating subjective sleepiness precisely would seem to be a visual analogue scale of sleepiness, rather than the SSS [48].

There remains an important question to be addressed about the relationship between the ability to overcome sleepiness and the attention-control function, which is regarded as one of the core functions of WM. Most studies have indicated a robust structure-function relationship between the left DLPFC and attention-control function [49]–[51]. Laterality discrepancy in the DLPFC between the ability to overcome sleepiness and attention-control may suggest a qualitative difference between them in executing WM processes.
